# Proteome‐Wide Analysis of Palmitoylated Proteins in Macrophages Reveals Novel Insights Into Early Immune Signaling

**DOI:** 10.1002/pmic.70100

**Published:** 2025-12-28

**Authors:** Hyojung Kim, Jiraphorn Issara‐Amphorn, Sung Hwan Yoon, Anirban Banerjee, Aleksandra Nita‐Lazar

**Affiliations:** ^1^ Functional Cellular Networks Section Laboratory of Immune System Biology National Institute of Allergy and Infectious Diseases, NIH Bethesda Maryland USA; ^2^ Section on Structural and Chemical Biology Neurosciences and Cellular and Structural Biology Division Eunice Kennedy Shriver National Institute of Child Health and Human Development NIH Bethesda Maryland USA

**Keywords:** acyl‐biotin exchange (ABE), immune signaling, lipopolysaccharide (LPS), macrophage, multi‐protease digestion, palmitoylation, posttranslational modification, proteomics, S‐acylation

## Abstract

Protein S‐palmitoylation, a reversible lipid modification, plays critical roles in regulating protein function and localization. However, its comprehensive role in the rapid reprogramming of macrophages during early immune responses remains incompletely understood. This study investigates the dynamics of the palmitoylome in immortalized bone marrow‐derived macrophages (iBMDMs) during the initial phase of lipopolysaccharide (LPS) stimulation. Employing acyl‐biotin exchange (ABE) proteomics coupled with a multi‐protease digestion strategy (trypsin, AspN, chymotrypsin, or GluC), we significantly enhanced palmitoylation proteome coverage, identifying 2502 putative S‐palmitoylated proteins (Log_2_ fold change > 2, FDR < 0.05). Notably, this approach uncovered 527 proteins not previously associated with the mouse palmitoylome, including 185 candidates exclusively identified using non‐tryptic proteases. In the context of immune cells, this study revealed 1378 proteins not previously reported, with 556 candidates identified exclusively via AspN, chymotrypsin, and/or GluC. Several of these novel candidates are established immune system components and phosphoproteins. Upon stimulation with 100 ng/mL LPS for 30 min, quantitative comparison revealed 648 differentially enriched proteins (308 predominantly detected in untreated, 340 predominantly detected in LPS‐treated), indicating dynamic regulation via this posttranslational modification during early innate immune activation. Functional enrichment analysis linked these dynamically regulated proteins to critical pathways: LPS treatment enriched for immune signaling cascades and infection pathways, while untreated cells showed enrichment for metabolic and transport processes. This study provides a comprehensive resource of the macrophage palmitoylome and its dynamic remodeling, offering novel targets for investigating the regulation of macrophage function.

## 1 Introduction

**FIGURE 1:**
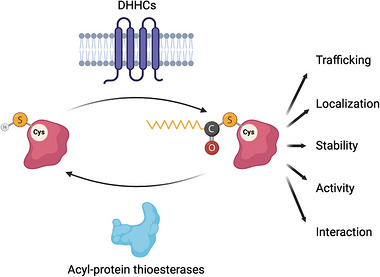
**T**he cycle of protein S‐palmitoylation and its functional consequences. DHHC enzymes add palmitate, influencing protein trafficking, localization, stability, activity, and interactions, while acyl‐protein thioesterases (APTs) remove the modification.

Macrophages are sentinel cells of the innate immune system, essential for tissue homeostasis, pathogen clearance, and orchestrating inflammatory response [1, 2]. Upon encountering stimuli such as bacterial lipopolysaccharide (LPS), macrophages undergo rapid and extensive reprogramming involving signaling cascades, transcriptional changes, and metabolic shifts to mount an effective immune response [3, 4]. Posttranslational modifications (PTMs) like phosphorylation, ubiquitination, and acetylation are well‐established key regulators of these processes. Protein S‐palmitoylation, the reversible covalent attachment of the 16‐carbon fatty acid palmitate to cysteine residues via a thioester bond (Figure 1), is an increasingly recognized PTM critical for regulating diverse cellular processes [5, 6]. It modulates protein hydrophobicity, influencing membrane association, subcellular localization, protein stability, protein–protein interactions, and vesicular trafficking [5]. While studies have identified palmitoylated proteins in various cell types and contexts, including immune cells [7], a comprehensive characterization of the palmitoylome and its dynamic changes during the critical early phase of macrophage activation by potent stimuli like LPS has been lacking. Understanding these early events is crucial as they set the stage for the subsequent, more sustained immune response.

Identifying palmitoylated proteins presents technical challenges for conventional proteomics. Palmitoylation is a labile modification in the cellular context, being reversed by the action of cellular thioesterases. It also lacks a consensus sequence, often occurring on membrane‐proximal, cytosol‐facing cysteines within hydrophobic regions, which can hinder detection by mass spectrometry [8, 9, 10, 11]. Current knowledge of the mouse immune palmitoylome is captured in databases such as the recently updated SwissPalm, which organizes information from large‐scale and targeted experiments [12]. In the immune context, SwissPalm lists 1641 non‐redundant mouse proteins derived from 7 large‐scale and 16 targeted experiments [13, 14, 15, 16, 17, 18, 19, 20, 21, 22, 23, 24, 25, 26, 27, 28, 29, 30, 31, 32, 33, 34, 35]. However, these datasets predominantly rely on the RAW 264.7 cell line [15, 23, 32, 35]. Reliance on this cell line may be limited to capture the physiology of primary immune cells. Furthermore, previous studies often employed longer LPS stimulation times (e.g., 1 h or more), potentially missing onset signaling events, and frequently relied solely on trypsin digestion. Trypsin digestion alone can limit sequence coverage, particularly for hydrophobic, membrane‐associated proteins that are common among palmitoylation [36].

This study aimed to overcome these limitations and provide a deeper, more comprehensive view of the macrophage palmitoylome during the initial phase of activation. We employed acyl‐biotin exchange (ABE) [37, 38, 39, 40] coupled with liquid chromatography‐tandem mass spectrometry (LC‐MS/MS) using immortalized bone marrow‐derived macrophages (iBMDMs), which closely mimic primary macrophage physiology, and a brief 30‐minute LPS stimulation [41, 42]. Critically, we utilized a multi‐protease digestion strategy (trypsin, AspN, chymotrypsin, GluC) to maximize protein and peptide identification, addressing the coverage limitations of trypsin. We hypothesized that this combined approach would reveal novel palmitoylated proteins and uncover dynamic changes in palmitoylation status upon short‐term LPS stimulation in iBMDMs. Our findings significantly expand the known macrophage palmitoylome and reveal widespread, dynamic regulation of palmitoylation within minutes of LPS exposure, highlighting its potential importance in shaping the initial immune response.

## 2 Materials and Methods

### 2.1 J2 Virus Containing Media Collection

J2 was a generous gift from Dr. Howard Young (National Cancer Institute, National Institutes of Health). J2 cells were seeded onto 15 cm cell culture dishes containing 20 mL of complete Dulbecco's Modified Eagle Medium (cDMEM), consisting of DMEM (Gibco, Cat no: 11995065) supplemented with 10% Fetal Bovine Serum (FBS) (Gibco) and maintained for 2 days. The supernatant was collected and centrifuged at 400 × *g* to remove cellular debris. The virus‐containing media from J2 cells was then filtered through a 0.45 µm filter before use.

### 2.2 Generation of Immortalized Bone Marrow‐Derived Mouse Macrophages (IBMDMs)

IBMDMs were generated from bone marrow‐derived macrophages (BMDMs), it is based on the protocol as previously described protocol [41]. Briefly, C57BL/6 mice (8–12 weeks old) were obtained from The Jackson Laboratory (https://www.jax.org/strain/000664) and maintained under pathogen‐free conditions at an American Association for the Accreditation of Laboratory Animal Care accredited animal facility at the NIAID and housed in accordance with the procedures outlined in the Guide for the Care and Use of Laboratory Animals under an animal study proposal approved by the NIAID Animal Care and Use Committee (protocol LISB17E). BMDMs were generated from bone marrow progenitor stem cells cultured in differentiation media containing DMEM (Gibco), supplemented with 10% fetal bovine serum (FBS) (Gibco), 5% horse serum (Gibco), and 25 ng/mL recombinant M‐CSF (R&D Systems). On day 4 of differentiation, 3 mL of fresh differentiation media were added to the cultures. On day 7, the culture media were replaced with a 1:1 mixture of differentiation media and J2 supernatant for 48 h. The virus‐containing media were then removed and replaced with fresh media containing M‐CSF, and cells were cultured for an additional week. The concentration of M‐CSF was slowly reduced over the following two weeks. Non‐immortalized cells gradually underwent cell death, while the remaining cells proliferated and grew in complete DMEM (cDMEM) without M‐CSF. The IBMDMs were subsequently characterized for marker expression and cellular phenotype.

### 2.3 Cell Culture and Stimulation

LPS derived from Salmonella typhimurium, TLR grade (ENZO life science) were used for IBMDMs stimulation. Briefly, LPS were diluted in cDMEM before adding to IBMDMs for 30 min (final concentration 100 ng/mL). Cell was washed twice with cold Phosphate Buffer Saline (PBS) before adding lysis buffer.

### 2.4 ABE Proteomics

ABE was performed based on established protocols with modifications [39, 40]. Briefly, cell pellets were lysed in Lysis Buffer (50 mM HEPES pH 7.4, 150 mM NaCl, 1% Igepal CA‐630, 0.1% SDS supplemented with protease inhibitors (Roche cOmplete). To prevent potential false positives arising from disulfide bond reduction during subsequent steps, proteins were first reduced by 50 mM tris(2‐carboxylethyl)phosphine (TCEP) for 1 h at RT. Excess TCEP was removed by protein precipitation by methanol/chloroform precipitation. The protein pellet was resuspended in Resuspension Buffer (2% SDS, 50 mM Tris‐HCl pH 7.4, 5 mM EDTA). Free thiol groups including those newly exposed by TCEP reduction were blocked with N‐ethylmaleimide (NEM, 50 mM) for 1 h at RT with gentle agitation. Excess NEM was removed by protein precipitation by methanol/chloroform precipitation. The protein pellet was resuspended in Resuspension Buffer. To ensure complete blocking of free thiol groups, NEM blocking was performed again following the same procedure [40]. After resuspension, samples were divided into two aliquots. Thioester bonds were cleaved, and newly exposed thiols were simultaneously biotinylated by treating one aliquot with Exchange Buffer (1 M hydroxylamine (HA), 1 mM HPDP‐biotin, 50 mM Tris‐HCl) for 1 h at room temperature. The control aliquot was treated with a control buffer (1 mM HPDP‐biotin, 50 mM Tris‐HCl, no HA) under identical conditions. Excess biotin reagent was removed by two rounds of methanol/chloroform protein precipitation to ensure complete removal of free biotin. Biotinylated proteins were enriched using streptavidin agarose beads (Thermo Fisher Scientific) by incubating lysate with beads for strictly 1 h at room temperature (RT) to minimize non‐specific binding. Beads were washed six times with Wash Buffer (0.1% SDS, 50 mM Tris‐HCl pH 7.4, 150 mM NaCl, 5 mM EDTA). For each wash, 600 µL of buffer was added, the resin was inverted 10 times, and beads were collected by centrifugation at 100 × *g* for 10 s at RT. Enriched proteins were eluted from the beads using Elution Buffer (0.1% SDS, 50 mM Tris‐HCl pH 7.4, 150 mM NaCl, 5 mM EDTA, 50 mM TCEP) to reduce the disulfide bond in the HPDP‐biotin linker, releasing the proteins with a free thiol at the original palmitoylation site. Following elution, these newly exposed thiols were alkylated by treatment with 50 mM iodoacetamide (IAA) for 30 min at RT in the dark to form stable carbamidomethyl adducts.

### 2.5 Western Blotting Validation

To validate the S‐palmitoylation of specific candidates, ABE was performed as described above up to the elution step. Following the streptavidin agarose enrichment and extensive washing, palmitoylated proteins were eluted from the beads using Elution Buffer (0.1% SDS, 50 mM Tris‐HCl pH 7.4, 150 mM NaCl, 5 mM EDTA, 50 mM TCEP). The eluates were mixed with 4× Laemmli sample buffer and incubated at room temperature for 15 min without boiling to prevent aggregation of hydrophobic membrane proteins. Proteins were separated by SDS‐PAGE and transferred onto PVDF membranes. Membranes were blocked with 5% non‐fat dry milk in TBST and incubated overnight at 4°C with primary antibodies against CPSF6 (Cell Signaling Technology, 92879) and GYG1 (Proteintech, 12836‐1‐AP). After washing, membranes were incubated with HRP‐conjugated secondary antibodies and visualized using enhanced chemiluminescence. Specific S‐palmitoylation was assessed by comparing the signal in hydroxylamine‐treated (HA+) samples versus hydroxylamine‐omitted (HA−) controls.

### 2.6 Protein Digestion Strategy

Eluted and alkylated proteins from both HA‐treated (test) and HA‐untreated (control) samples were processed for digestion. To enhance coverage, parallel digestions were performed. Aliquots of the enriched proteins were subjected to in‐solution digestion for 18 h using sequencing‐grade modified proteases: trypsin (Promega), AspN (Roche), chymotrypsin (Promega), and GluC (Promega). Accurately quantifying the low protein amounts after enrichment and prior to digestion was challenging; measurement by nanodrop at this stage was precluded by the complex buffer composition (nanodrop was utilized later, after digestion and desalting). Based on previous experience and post‐digestion quantification attempts, the yield was estimated to be 100 µg or less protein per sample starting from 2 mg initial lysate. Therefore, an approximate enzyme:protein ratio of 1:100 (w/w) was used for all enzymes, based on this estimated protein amount. Digestions with trypsin, AspN, and GluC were performed at 37°C, while chymotrypsin digestion was performed at 25°C. Digestions were carried out in 50 mM ammonium bicarbonate.

### 2.7 Mass Spectrometry for ABE With Trypsin

Thermo Orbitrap Q‐Exactive HF (Thermo Fisher Scientific, Bremen, Germany) and Thermo UltiMate 3000 (Thermo Fisher Scientific, Bremen, Germany) were used for LC‐MS/MS experiments. Peptides were trapped on a Acclaim C18 PepMap 100 trap column (5 µm, 100 Å, 300 µm i.d. × 5 mm, Thermo Fisher Scientific, Pittsburgh, PA) and separated on a PepMap RSLC C18 column (2 µm, 100 Å, 75 µm i.d. × 50 cm, Thermo Fisher Scientific, Pittsburgh, PA) at 35°C. The LC steps were: 98% mobile phase A (0.1% v/v formic acid in H_2_O) and 2% mobile phase B (0.1% v/v formic acid in ACN) from 0 to 5 min, 2% to 35% linear gradient of mobile phase B from 5 to 155 min, 35% to 85% linear gradient of mobile phase B from 155 to 157 min, 85% mobile phase B from 157 to 170 min, 85% to 2% linear gradient of mobile phase B from 170 to at 172 min, 2% of mobile phase B from 172 to 190 min. Eluted peptides were ionized in positive ion polarity at a 2.3 kV of spraying voltage. MS^1^ full scans were recorded in the range of m/z 350 to 1700 with a resolution of 60,000 at 200 m/z using the Orbitrap mass analyzer. Automatic gain control was set at 3 × 10^6^ with 60 ms of maximum injection time. Top 20 data dependent acquisition mode was used to maximize the number of MS2 spectra from each cycle. Higher‐energy collision‐induced dissociation (HCD) was used to fragment selected precursor ions with a normalized collision energy of 27%.

### 2.8 Mass Spectrometry for ABE With AspN, Chymotrypsin, or GluC

A Thermo Orbitrap Fusion Eclipse (Thermo Fisher Scientific, San Jose, USA) coupled to a Thermo UltiMate 3000 (Thermo Fisher Scientific) was used for LC‐MS/MS experiments. One microgram of total peptides were injected for LC‐MS/MS analysis. Peptides were trapped on an Acclaim C18 PepMap 100 trap column (5 µm particles, 100 Å pores, 300 µm i.d. × 5 mm, Thermo Fisher Scientific) and separated on a PepMap RSLC C18 column (2 µm particles, 100 Å pores, 75 µm i.d. × 50 cm, Thermo Fisher Scientific) at 40°C. The LC steps were: 98% mobile phase A (0.1% v/v formic acid in H_2_O) and 2% mobile phase B (0.1% v/v formic acid in ACN) from 0 to 5 min, 2% to 35% linear gradient of mobile phase B from 5 to 155 min, 35% to 85% linear gradient of mobile phase B from 155 to 157 min, 85% mobile phase B from 157 to 170 min, 85% to 2% linear gradient of mobile phase B from 170 to at 172 min, 2% of mobile phase B from 172 to 190 min. Eluted peptides were ionized in positive ion polarity at a 2.1 kV of spraying voltage. MS^1^ full scans were recorded in the range of m/z 375 to 1500 with a resolution of 120,000 at 200 m/z using the Orbitrap mass analyzer. Automatic gain control and maximum injection time were set to standard and auto, respectively. Top 3 s data dependent acquisition mode was used to maximize the number of MS^2^ spectra from each duty cycle. HCD was used to fragment selected precursor ions with normalized collision energy of 27. MS^2^ scans were recorded using an automatic scan range with a resolution of 15,000 at 200 m/z using the Orbitrap mass analyzer.

### 2.9 Data Analysis

Raw mass spectrometry data files were processed using MaxQuant software (2.4.14.0) [43, 44]. Peptide and protein identification was performed using the integrated Andromeda search engine searching against the UniProt mouse reference proteome database (UP000000589) combined with a database of common contaminants. Search parameters included: enzyme specificity set for trypsin, AspN, chymotrypsin, or GluC, allowing up to two missed cleavages for trypsin and three for the others; precursor mass tolerance of 20 ppm first search, 4.5 ppm main search; fragment ion mass tolerance of 20 ppm; variable modifications: Oxidation (M), Acetyl (Protein N‐term), NEM (C), Carbamidomethyl (C), and Deamidation (NQ). Separate label‐free quantification (LFQ) was enabled for each HA+ and HA− group. Peptide and protein identifications were filtered based on a false discovery rate (FDR) of < 1% determined by the target‐decoy approach.

### 2.10 Statistical Analysis

To identify putative palmitoylated proteins, enrichment analysis was performed. Missing values in the LFQ intensity matrices for the HA+ and HA− groups were imputed separately within each group using minimum value imputation from a normal distribution (width 0.3, downshift 1.8), simulating low abundance proteins [45]. Subsequently, the imputed LFQ intensities from HA‐treated samples (HA+) were compared to those from control (HA−) samples across replicates using Welch's *t*‐test. Proteins were considered palmitoylation candidates if they showed statistically significant enrichment (FDR < 0.05, calculated using Benjamini‐Hochberg and a Log_2_ fold change (FC) > 2 calculated based on the ratio of means of imputed and log_2_ transformed LFQ intensities between HA+ and HA− groups. While imputation allowed for finite fold‐change calculation for all proteins, confidence levels were assigned based on the calculated Log_2_ fold change (Log_2_ FC > 2: Low Confidence; Log_2_ FC > 4: Medium Confidence) or the original detection pattern (exclusive detection in HA+ replicates prior to imputation: High Confidence). To assess changes upon LPS stimulation, proteins predominantly detected or significantly enriched in either the untreated or LPS‐treated condition were considered. Functional annotation and enrichment analysis of identified and dynamically regulated proteins were performed using the STRING database (12.0) [46]. Data processing, statistical analysis, and data visualization were conducted using Python (3.9) utilizing standard scientific libraries.

**FIGURE 2:**
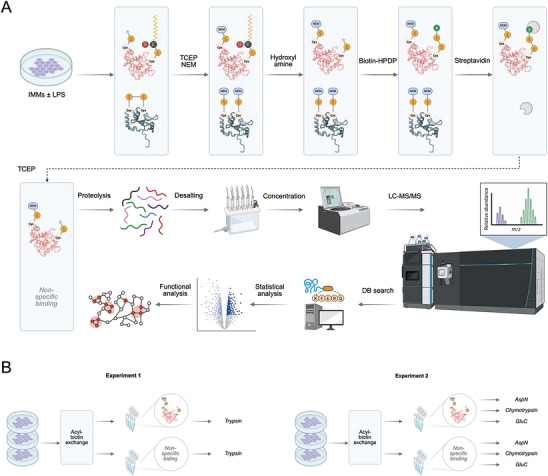
Experimental workflow for proteome‐wide analysis of S‐palmitoylation in macrophages. IMMs were cultured with or without LPS stimulation. Proteins were extracted, subjected to Acyl‐Biotin Exchange (ABE) involving TCEP reduction, NEM blocking, hydroxylamine cleavage/biotinylation, and streptavidin enrichment. Enriched proteins were digested using multiple proteases (trypsin for Exp. 1 and AspN, chymotrypsin, or GluC for Exp. 2), analyzed by LC‐MS/MS, and data were processed using MaxQuant followed by statistical and functional analysis.

## 3 Results

### 3.1 Global Proteome Stability During Early LPS Stimulation

**FIGURE 3:**
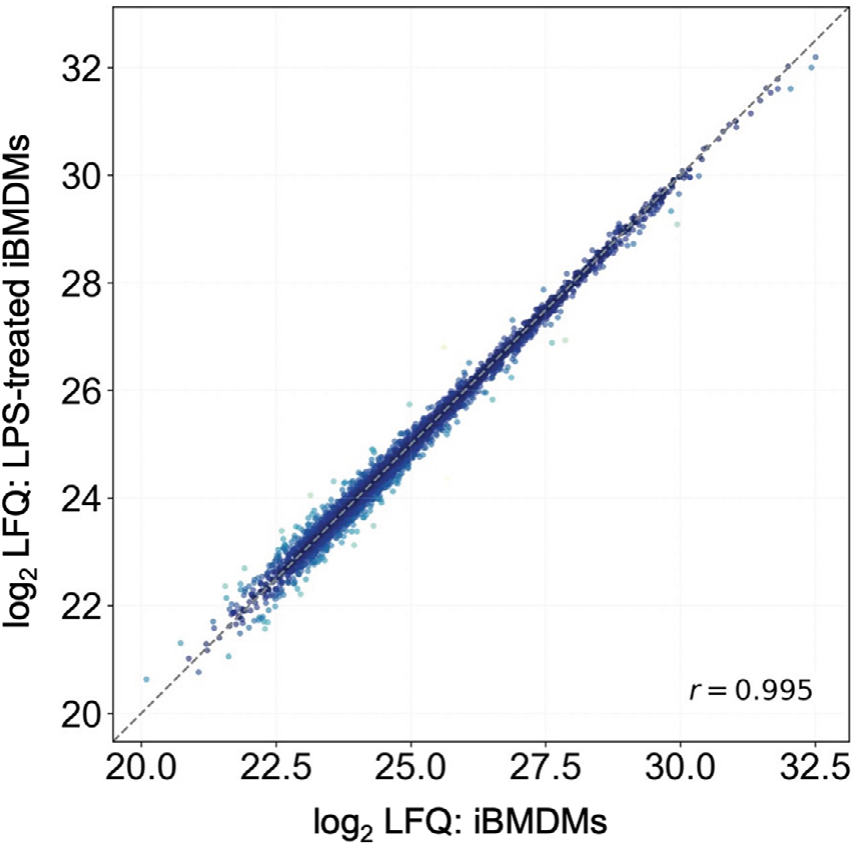
Global proteome stability after short‐term LPS stimulation. Scatter plot comparing Log2 LFQ intensities of proteins identified in untreated IMMs (x‐axis) versus LPS‐treated IMMs (y‐axis). Each point represents a protein. The high Pearson correlation coefficient (*r* = 0.995) indicates minimal change in overall protein abundance after 30 min of LPS treatment.

To assess the overall effect of the 30‐minute LPS incubation on global protein expression, we compared the proteomic profiles of untreated iBMDMs and those stimulated with LPS (*n* = 3 biological replicates per group). Correlation analysis of Log_2_ LFQ intensities per protein between the conditions showed a strong linear relationship (Pearson correlation coefficient, *r* = 0.995), indicating that overall protein abundances were not significantly affected by the short LPS stimulation (Figure 3). This suggests that changes observed in the ABE‐enriched fraction primarily reflect alterations in S‐palmitoylation status rather than major shifts in total protein levels.

### 3.2 Comprehensive Identification of Palmitoylated Proteins in Macrophages Using Multi‐Protease Strategy

**FIGURE 4:**
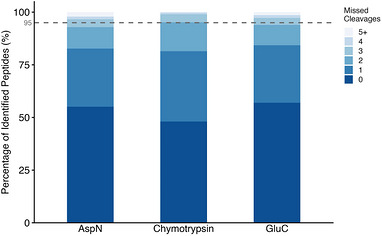
Determination of optimal missed cleavages for non‐tryptic proteases. Stacked bar charts show the cumulative percentage of identified peptides (y‐axis) obtained with increasing numbers of allowed missed cleavages up to 10 for AspN, chymotrypsin, and GluC digests. The dashed line indicates the 95% threshold. Allowing three missed cleavages achieved > 95% coverage for all three enzymes (AspN: 96.6%, chymotrypsin: 99.0%, GluC: 97.3%).

To comprehensively map the macrophage palmitoylome, we employed ABE enrichment followed by LC‐MS/MS analysis using four distinct proteases (trypsin, AspN, chymotrypsin, or GluC) (Figure 2). We first determined the optimal number of allowed missed cleavages for the non‐tryptic proteases. By analyzing the MS data allowing up to 10 missed cleavages with default MaxQuant settings for efficiency, we found that allowing three missed cleavages was sufficient to identify > 95% of the peptides captured with 10 allowed missed cleavages for AspN (96.6%), Chymotrypsin (99.0%), and GluC (97.3%) (Figure 4). Therefore, three missed cleavages were used for subsequent analyses.

### 3.3 Identification of Novel Palmitoylation Candidates

**FIGURE 5:**
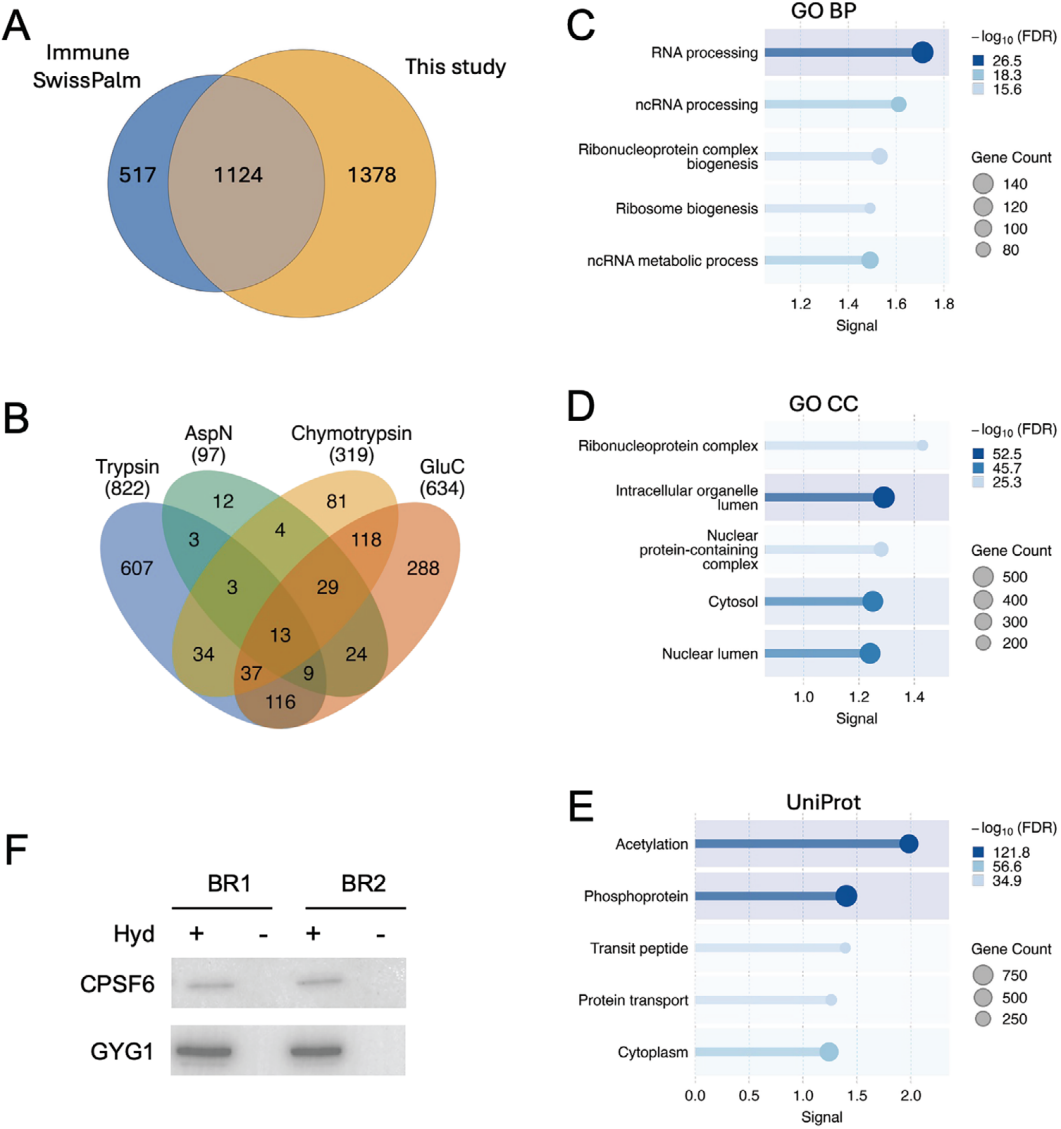
Identification and characterization of novel palmitoylation candidates in macrophages. (A) Venn diagram comparing the S‐palmitoylated proteins identified in this study (2502 total) with the Mouse Immune SwissPalm database. The overlap indicates previously reported candidates, while 1378 proteins (orange) represent novel candidates identified in this study. (B) Venn diagram showing the contribution of each protease (trypsin, AspN, chymotrypsin, GluC) to the identification of the 1378 novel proteins. Numbers indicate proteins identified exclusively by a specific protease or shared between them. (C–E) Functional enrichment analysis of the 1378 novel candidates. Dot plots display the top significantly enriched terms for (C) GO Biological Process, (D) GO Cellular Component, and (E) UniProt Annotation Keywords. The x‐axis (Signal) represents a weighted harmonic mean of the observed/expected ratio and ‐log_10_ FDR, designed to balance enrichment magnitude and statistical significance. The size of the dot corresponds to the number of genes (Gene Count), and the color intensity represents the significance (‐log_10_ FDR). (F) Validation of novel palmitoylation candidates. ABE enrichment followed by western blotting confirmed the S‐palmitoylation of endogenous CPSF6 and GYG1 in iBMDMs. The presence of a band in the hydroxylamine‐treated sample (Hyd+) compared to the control (Hyd−) indicates specific S‐palmitoylation.

To evaluate the novelty of our dataset, we first compared our identified S‐palmitoylated proteins to the entire mouse section of the SwissPalm database. This global comparison revealed 527 proteins that were not previously documented as palmitoylated in any mouse tissue, representing a significant expansion of the known species‐wide palmitoylome (Figure S1A). We then refocused our analysis on the “Mouse Immune” subset of SwissPalm to assess novelty specifically within the immune context. This comparison highlighted an even deeper coverage gap: of the 2502 proteins confidently identified in this study, 1378 were not previously reported in the Mouse Immune SwissPalm database, representing novel palmitoylation candidates in the context of immune cells (Figure 5A). The identification of these novel candidates was significantly driven by our multi‐protease strategy. As shown in the protease overlap analysis of these 1378 proteins, while trypsin identified a large portion (822 proteins total, 607 exclusively), a substantial fraction (556 proteins) was identified exclusively by non‐tryptic proteases (AspN, chymotrypsin, or GluC) or combinations thereof (Figure 5B). This confirms that expanding digestion beyond trypsin is essential for capturing the full extent of the palmitoylome. To characterize the biological functions and localization of these novel candidates, we performed Gene Ontology (GO) and UniProt Annotation Keywords enrichment analyses. GO Biological Process (BP) analysis revealed that these proteins are heavily involved in “RNA processing,” “ncRNA metabolic process,” and “Ribonucleoprotein complex biogenesis” (Figure 5C). Consistent with this, GO Cellular Component (CC) analysis showed strong enrichment for “Ribonucleoprotein complex,” “Intracellular organelle lumen,” and “Nuclear protein‐containing complex” (Figure 5D). Furthermore, UniProt Annotation Keywords analysis highlighted “Phosphoprotein” and “Acetylation” as the most significant features (Figure 5E), suggesting a high prevalence of co‐occurring PTMs among these novel palmitoylation targets. To validate these findings, we selected two candidates from the novel list, CPSF6 (identified with GluC digest) and GYG1 (identified with GluC, AspN, and chymotrypsin), which are known phosphoproteins involved in RNA processing and metabolism, respectively. Using ABE coupled with western blotting, we successfully confirmed the S‐palmitoylation of both endogenous CPSF6 and GYG1 in iBMDMs (Figure 5F), supporting the reliability of our large‐scale MS identification.

### 3.4 Dynamic Remodeling of the Macrophage Palmitoylome Upon LPS Stimulation

**FIGURE 6:**
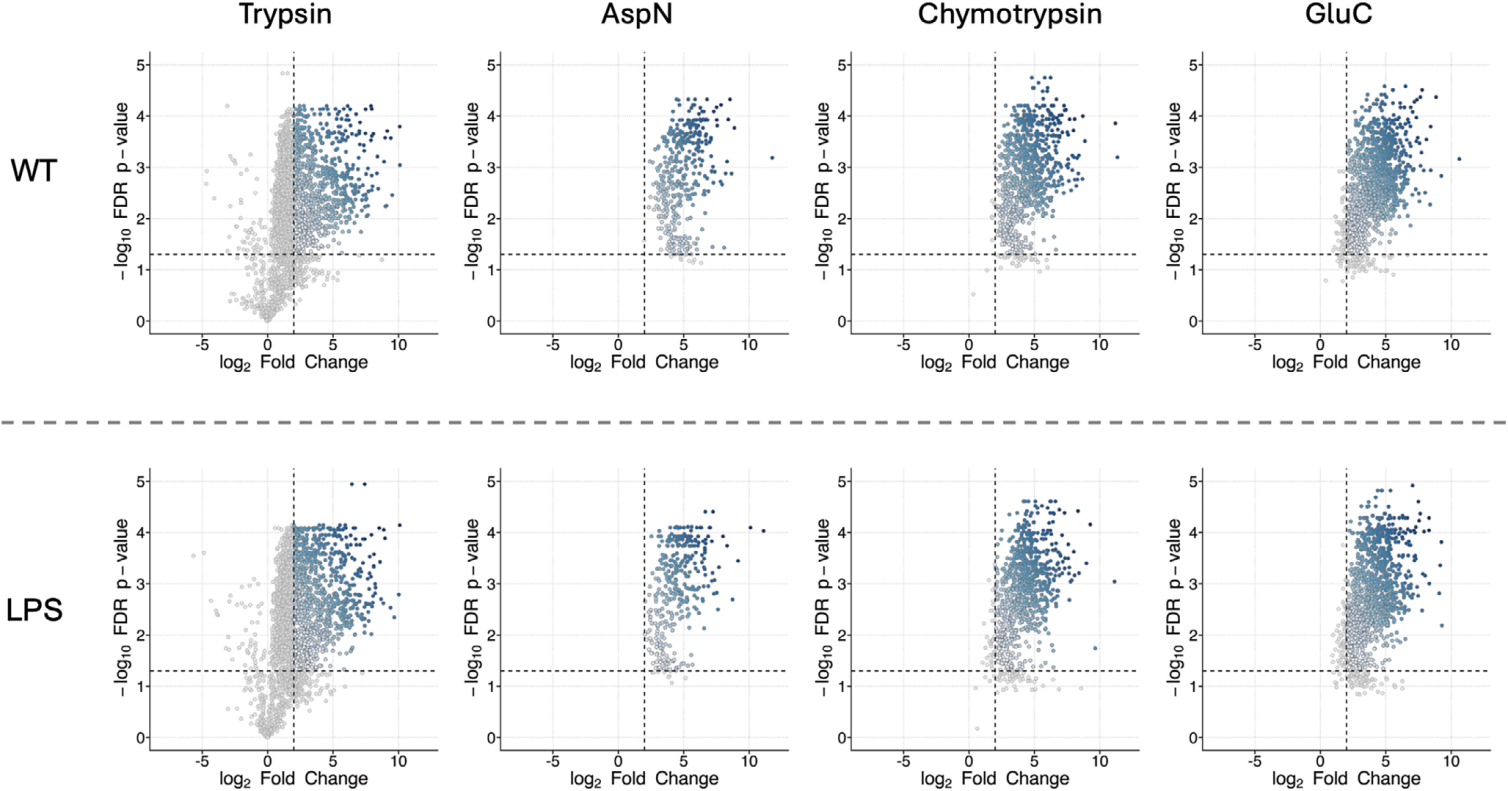
Volcano plots showing enrichment of palmitoylated proteins by ABE. Plots compare HA‐treated (HA+) versus HA‐untreated (HA−) control samples for each protease digest in untreated (WT, top row) and LPS‐stimulated (LPS, bottom row) conditions. Proteins are plotted by Log_2_ (Fold Change HA±HA) (x‐axis) versus ‐Log_10_ (FDR *p* value) (y‐axis). Points colored blue/dark blue represent significantly enriched proteins (FDR < 0.05, Log_2_ FC > 2), indicating putative palmitoylation candidates.

**FIGURE 7:**
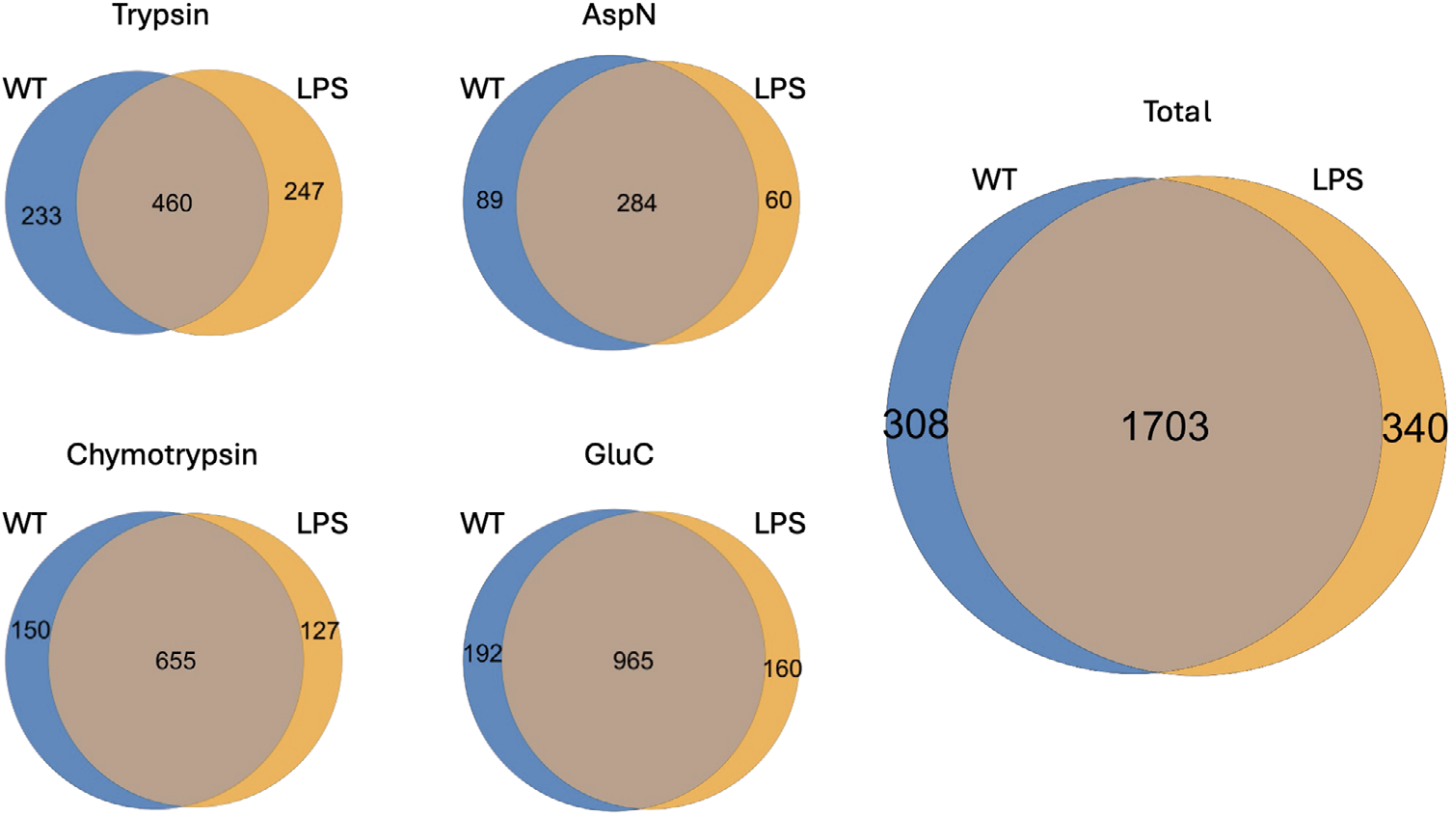
Comparison of identified palmitoylomes between untreated and LPS‐stimulated conditions. Venn diagrams show the overlap of confidently identified palmitoylated proteins (Log2FC > 2, FDR < 0.05) between untreated (WT) and LPS‐stimulated (LPS) samples. Comparisons are shown for each protease digest individually (trypsin, AspN, chymotrypsin, or GluC) and integrated across all proteases. Numbers indicate proteins detected exclusively in WT, exclusively in LPS, or in both conditions.

We next investigated how the palmitoylome changes during the early phase of macrophage activation (30 min LPS stimulation). Volcano plots comparing HA+ versus HA− samples for each protease and condition illustrate the enrichment of palmitoylated proteins (Figure 6). Proteins passing the significance threshold (FDR < 0.05) and enrichment cutoff (Log_2_ FC > 2) are highlighted in a gradient way. To assess dynamic changes upon LPS stimulation, we compared the sets of confidently identified palmitoylated proteins between the untreated (WT) and LPS‐treated conditions for each protease individually and in an integrated manner (Figure 7). Notably, the proteins assigned as high confidence were exclusively identified in HA+ samples and this is a common feature for the identified DHHCs in this study. DHHC enzymes catalyze S‐palmitoylation and undergo S‐palmitoylation at the active site as the first step of the catalysis. This they serve as the positive controls. So, we decided to focus on those types of proteins. The integrated analysis, combining data from all proteases, revealed 308 proteins predominantly detected in untreated cells and 340 proteins predominantly detected in LPS‐treated cells, totaling 648 proteins showing condition‐specific enrichment (Figure 7, Left). This indicates a rapid and substantial remodeling of the palmitoylome within minutes of LPS exposure.

### 3.5 Functional Annotation of Dynamically Regulated Palmitoylated Proteins

**FIGURE 8:**
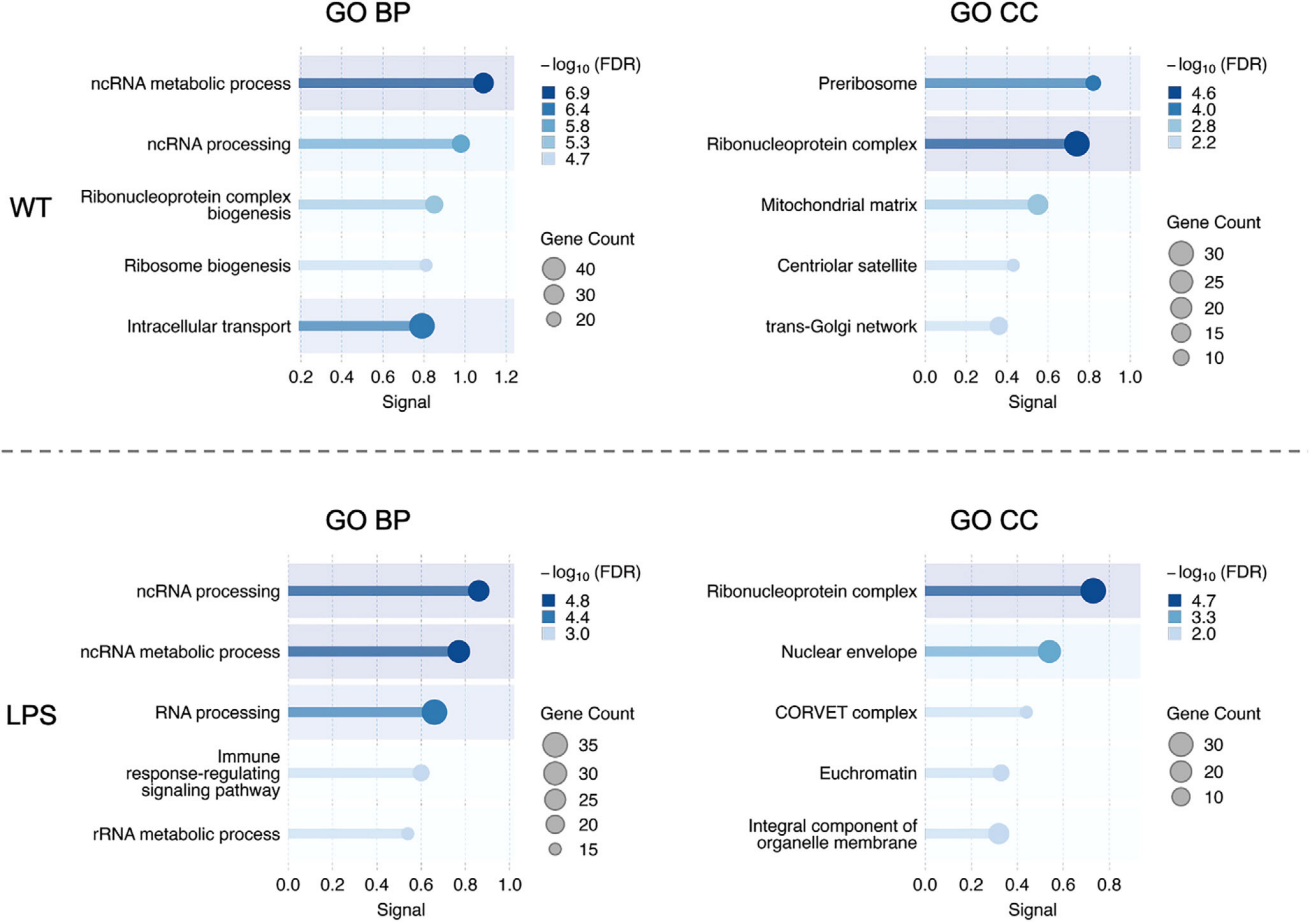
Gene ontology (GO) enrichment analysis of dynamically regulated palmitoylated proteins. Dot plots show the top significantly enriched GO terms for proteins predominantly detected in untreated WT cells (top, 308 proteins) or LPS‐treated cells (bottom, 340 proteins). Enrichment results are categorized into Biological Process (GO BP) and Cellular Component (GO CC). The x‐axis (Signal) represents a weighted harmonic mean of the observed/expected ratio and log_10_ FDR. The dot size corresponds to the number of genes (Gene Count), and the color intensity represents the significance (log_10_ FDR).

**FIGURE 9:**
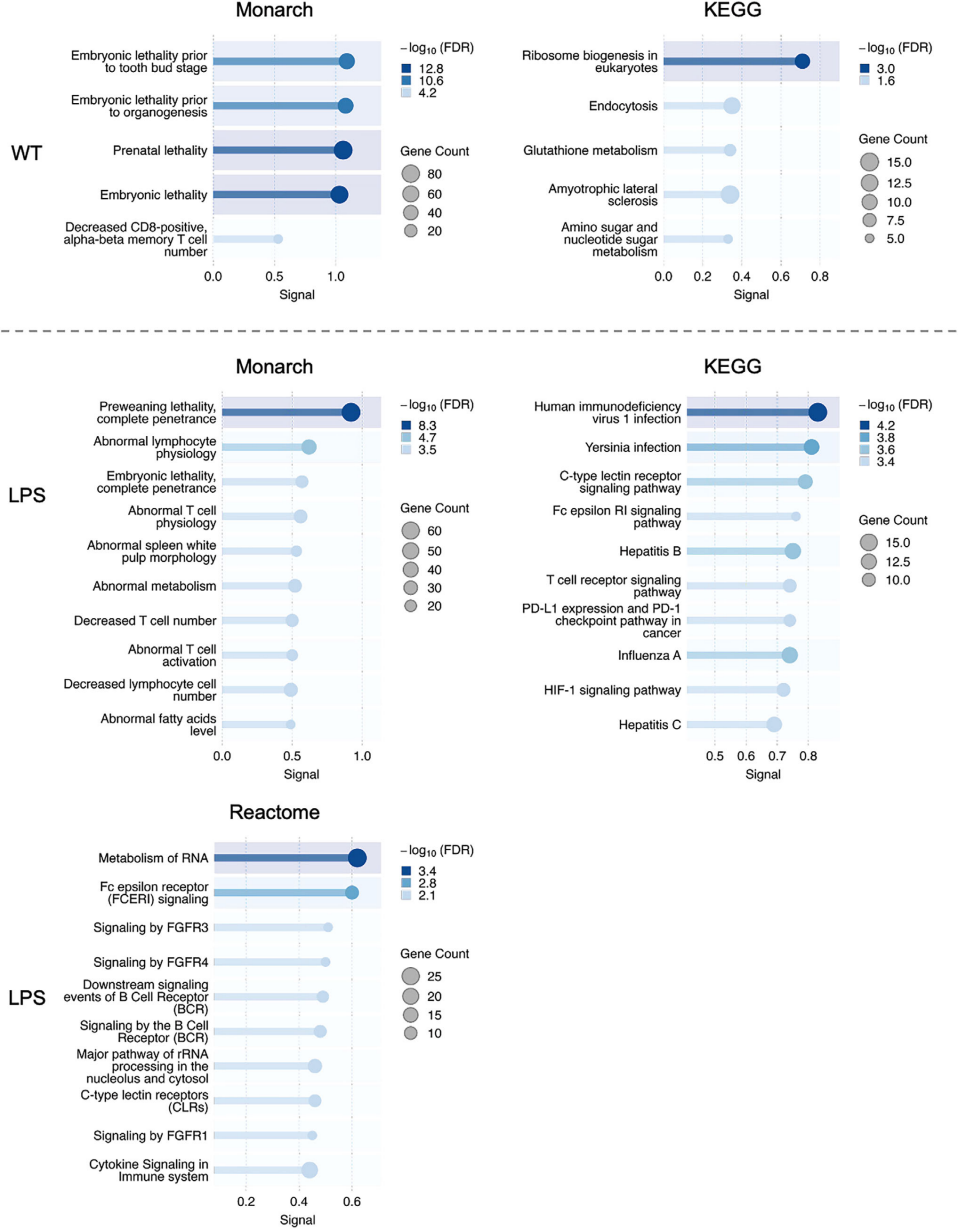
Pathway and phenotype enrichment analysis of dynamically regulated palmitoylated proteins. Dot plots display the enrichment results using Monarch (Mammalian Phenotype Ontology), KEGG Pathways, and Reactome Pathways databases. (Top) Enrichment for proteins predominantly detected in untreated WT cells (308 proteins) using Monarch and KEGG databases. (Bottom) Enrichment for proteins predominantly detected in LPS‐treated cells (340 proteins) using Monarch, KEGG, and Reactome databases. The x‐axis (Signal) represents a weighted harmonic mean of the observed/expected ratio and log_10_ FDR. The dot size corresponds to the gene count, and the color intensity represents the significance (log_10_ FDR).

To understand the biological significance of the rapid palmitoylome remodeling, we performed comprehensive functional enrichment analysis on the 648 proteins showing condition‐specific S‐palmitoylation. To focus on the most biologically relevant associations, we filtered for highly enriched terms across Gene Ontology (GO), Mammalian Phenotype Ontology (Monarch), KEGG Pathways, and Reactome Pathways databases. Proteins predominantly enriched in untreated cells (308 proteins) appeared to cluster around fundamental cellular maintenance and homeostasis. GO and pathway analyses pointed to a reliance on terms such as “ribosome biogenesis,” “RNA processing,” and metabolic processes like “glutathione metabolism” (Figures 8A,B and 9, Top). The appearance of phenotype terms related to “embryonic lethality” and “prenatal lethality” (Figure 9, Top) further suggests that constitutive palmitoylation may support essential core machinery required for macrophage viability and basic physiology in the resting state. In striking contrast, the functional profile of proteins enriched after LPS stimulation (340 proteins) shifted toward active host defense and immune signaling. This group showed significant enrichment for terms such as “immune system process” and “immune response‐regulating signaling pathway” (Figure 8C,D). Pathway analysis indicated that this pattern was not limited to a single cascade. Instead, it likely reflects a broad activation of antipathogen mechanisms as evidenced by the enrichment of multiple infection‐related pathways like Influenza, Tuberculosis, Yersinia, and HIV (Figure 9, Bottom). This implies that LPS stimulation might rapidly repurpose the palmitoylation machinery to target proteins conserved across various antimicrobial responses. Specific signaling modules were also heavily enriched in the LPS‐induced set. These included the “C‐type lectin receptor,” “Fc epsilon RI,” and “T cell receptor” signaling pathways (Figure 9, Bottom). This functional reorganization is consistent with the dynamic palmitoylation of key immune mediators detected in this group, such as MyD88, Map2k3, IRF9, and MARCKS. The detection of these signaling nodes suggests that their palmitoylation could facilitate their rapid recruitment to membrane microdomains, which potentially contributes to the orchestration of the inflammatory response.

## 4 Discussion

This study provides an extensive map of the macrophage S‐palmitoylome and identifies 2502 candidate proteins using a multi‐protease ABE strategy. This approach significantly expanded the known palmitoylome landscape. Compared to the existing Mouse Immune SwissPalm database, we uncovered 1378 novel candidates for palmitoylation in the context of the innate immune cells. The use of complementary proteases (AspN, chymotrypsin, and GluC) was crucial as it uniquely identified 556 of these novel proteins. These non‐tryptic candidates were notably enriched in phosphoproteins, which highlights the power of multi‐protease digestion for in‐depth PTM analysis and suggests a broad interplay between palmitoylation and phosphorylation in macrophages.

Our results demonstrate that protein palmitoylation is highly dynamic and undergoes substantial remodeling within just 30 min of LPS stimulation. We identified 648 proteins exhibiting significant changes in detection between untreated and LPS‐treated states (308 WT‐exclusive, 340 LPS‐exclusive). Given the stability of the global proteome within this timeframe (Figure 3), these changes likely reflect alterations in palmitoylation levels or protein localization influenced by palmitoylation rather than changes in protein expression.

Functional analysis of the dynamically regulated proteins offers insights into the role of palmitoylation in the early LPS response. The enrichment of immune signaling pathways, response to infection pathways, and related signaling terms among those predominantly detected after LPS treatment (340 proteins) aligns with the known roles of palmitoylation in promoting membrane association and facilitating signal transduction. This suggests that LPS triggers increased palmitoylation or recruitment of key inflammatory mediators to membrane microdomains.

Conversely, the enrichment of intracellular components, RNA processing, and metabolic process terms among proteins predominantly detected in the untreated state (308 proteins) suggests that LPS may induce de‐palmitoylation or relocalization of proteins involved in constitutive cellular processes. This could represent a mechanism to redirect resources or modulate receptor trafficking. Additionally, the enrichment of terms related to “lethality” and essential developmental processes in the untreated set warrants further exploration as it implies that basal palmitoylation is critical for macrophage viability and homeostasis. The identification of numerous novel palmitoylated proteins opens new research avenues. This is particularly true for those involved in immunity and signaling which were previously overlooked by trypsin‐only approaches. Understanding how palmitoylation regulates these specific proteins by affecting their localization, interactions, or stability is crucial for a deeper understanding of macrophage biology and innate immune regulation.

## 5 Limitations

The ABE method detects thioester linkages broadly, identifying candidates for S‐acylation which, while primarily S‐palmitoylation, could include other fatty acids. Secondly, the label‐free quantification approach measures relative enrichment, serving as a proxy for changes in palmitoylation state rather than providing direct stoichiometric information or definitively distinguishing between modification level changes. A related point concerns the interpretation of “predominantly detected” proteins, defined by thresholding after imputation; while useful for highlighting potential changes, this categorization might overstate differences for proteins near the detection limit. However, confidence in identifying biologically relevant changes, including those categorized as “predominantly detected,” is substantially increased by the successful and consistent high‐confidence enrichment of multiple DHHC palmitoyltransferases, known autopalmitoylated positive controls, which validates the core ABE procedure. Therefore, while the DHHC data support the overall validity, the specific limitations regarding S‐acylation specificity and the precise nature of the quantitative changes remain and are being addressed through the planned future directions using orthogonal methods.

## 6 Future Directions

Ongoing work involves using biorthogonal labeling with clickable fatty acid analogs such as 17‐Octadecynoic acid (17‐ODYA) coupled with click chemistry to validate key hits and potentially map modification sites. High‐interest candidates exhibiting dynamic changes, particularly novel immune‐related proteins, will be functionally validated using site‐directed mutagenesis (Cys‐to‐Ser mutants) and cell‐based assays (cytokine production, phagocytosis, signaling) to determine the functional consequences of their palmitoylation status during macrophage activation.

## 7 Conclusion

In summary, this study utilized an enhanced multi‐protease ABE proteomics workflow to generate a deep map of the macrophage palmitoylome, identifying 2502 candidate proteins, including 1378 candidates not previously reported in the immune context. We revealed significant and dynamic alterations in protein palmitoylation profiles within 30 min of LPS exposure, with 648 proteins showing differential enrichment between conditions, affecting key cellular pathways in immune signaling, transport, and organization. These findings highlight the critical role of dynamic S‐palmitoylation in orchestrating the early innate immune response and provide a valuable resource for future investigations into macrophage biology and immune regulation.

## Conflicts of Interest

The authors declare no conflicts of interest.

## Supporting information




**Supporting File 1**: pmic70100‐sup‐0001‐Tables.zip.

**Supporting File 2**: pmic70100‐sup‐0002‐FiguresS1‐S2.docx.

## Data Availability

The data that support the findings of this study are openly available in PRIDE at https://proteomecentral.proteomexchange.org, reference number PXD063463.
