# Supplementary material for: Proteome‐Wide Analysis of Palmitoylated Proteins in Macrophages Reveals Novel Insights Into Early Immune Signaling
Source: Proteomics. 2025 Dec 28;26(8):69–80. doi: 10.1002/pmic.70100 (PMC13419279; doi:10.1002/pmic.70100)
Supplement: Supplementary file 2 — Supporting File 2: pmic70100‐sup‐0002‐FiguresS1‐S2.docx. [file PMIC-26--s001.docx]

**Supporting Information**


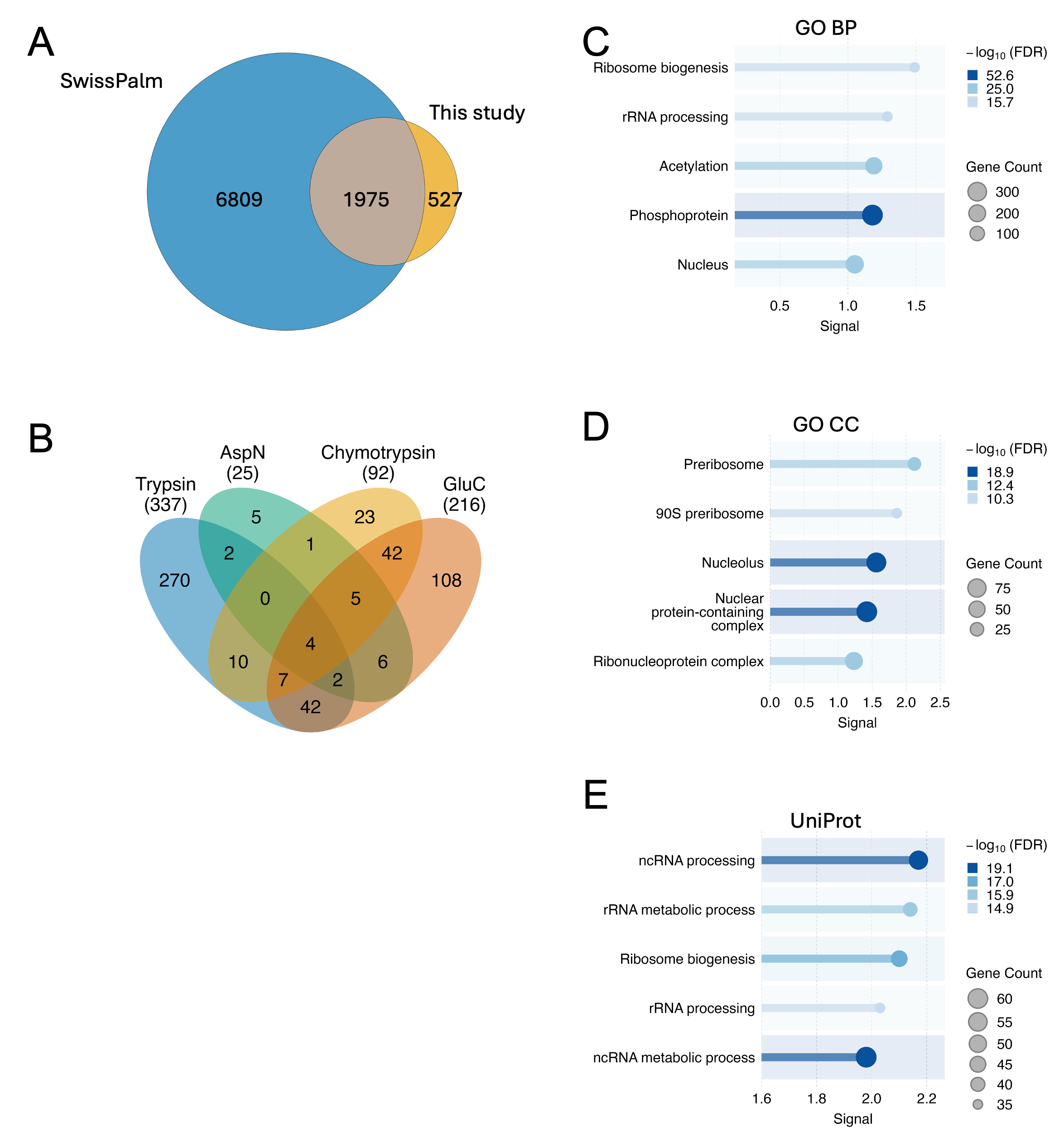


**Fig. S1. Identification and characterization of species-novel palmitoylation candidates.** **(A)** Venn diagram comparing the S-palmitoylated proteins identified in this study (2502 total) with the complete Mouse SwissPalm database (8784 proteins). The 527 proteins (orange) represent species-novel candidates not previously reported in any mouse tissue. **(B)** Venn diagram showing the contribution of each protease (Trypsin, AspN, Chymotrypsin, GluC) to the identification of the 527 species-novel proteins. Numbers in parentheses indicate the total number of novel proteins identified by that specific protease. **(C - E)** Functional enrichment analysis of the 527 species-novel candidates. Dot plots display the top significantly enriched terms for **(C)** GO Biological Process, **(D)** GO Cellular Component, and **(E)** UniProt Annotation Keywords. The x-axis (Signal) represents a weighted harmonic mean of the observed/expected ratio and -log_10_ FDR. The dot size corresponds to the gene count, and the color intensity represents the significance (-log_10_ FDR).


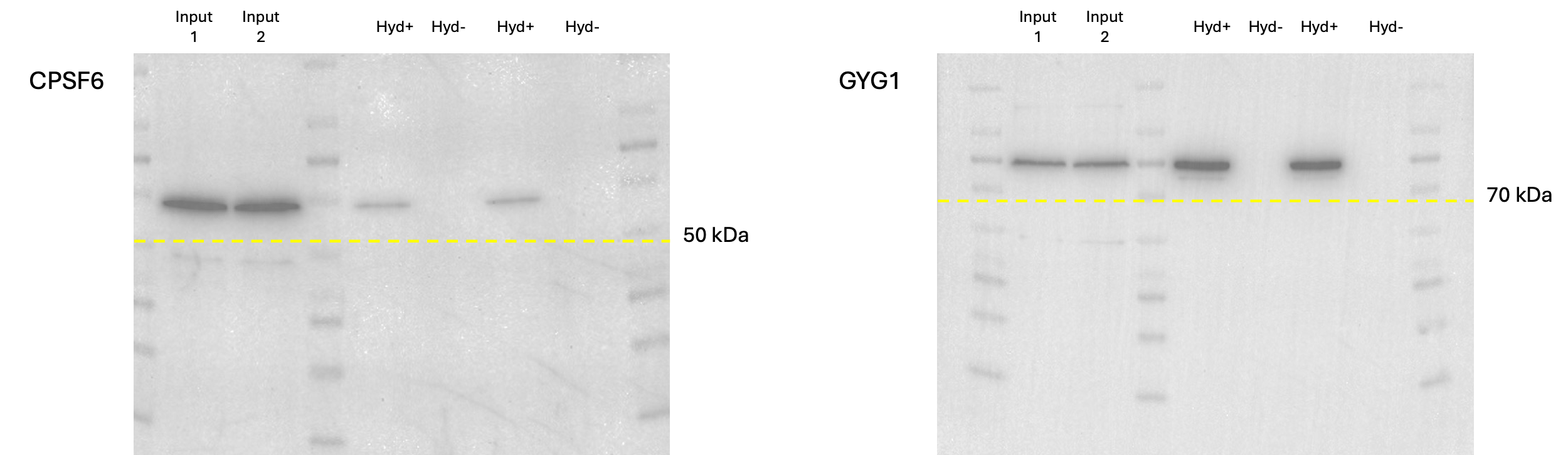


**Fig. S2. Validation of S-palmitoylation for CPSF6 and GYG1 by ABE-Western blotting.** Western blot analysis of CPSF6 (left) and GYG1 (right) following Acyl-Biotin Exchange (ABE) enrichment in iBMDMs. Input: Total cell lysate collected prior to streptavidin pull-down. Hyd+: Samples treated with hydroxylamine to cleave thioester bonds, resulting in the specific elution of S-palmitoylated proteins. Hyd-: Control samples treated with buffer (omitting hydroxylamine) to assess non-specific binding. The strong signal in Hyd+ lanes compared to the negligible signal in Hyd- lanes confirms the specific S-palmitoylation of both candidates. Note: Samples were prepared under mild conditions (no boiling) to preserve membrane protein integrity; consequently, GYG1 was detected primarily as a dimer (~70 - 80 kDa) consistent with its native quaternary structure. Yellow dashed lines indicate the position of molecular weight markers.
